# A Rare Variant in* PGAP2* Causes Autosomal Recessive Hyperphosphatasia with Mental Retardation Syndrome, with a Mild Phenotype in Heterozygous Carriers

**DOI:** 10.1155/2017/3470234

**Published:** 2017-10-08

**Authors:** Yonatan Perez, Ohad Wormser, Yair Sadaka, Ruth Birk, Ginat Narkis, Ohad S. Birk

**Affiliations:** ^1^The Morris Kahn Laboratory of Human Genetics, National Institute for Biotechnology in the Negev and Faculty of Health Sciences, Ben Gurion University of the Negev, 84105 Beer Sheva, Israel; ^2^Pediatric Neurology Unit, Division of Pediatrics, Soroka University Medical Center, Faculty of Health Sciences, Ben Gurion University of the Negev, 84101 Beer Sheva, Israel; ^3^Faculty of Health Sciences, Ariel University, Ariel, Israel; ^4^Genetics Institute, Soroka University Medical Center, Ben Gurion University of the Negev, 84101 Beer Sheva, Israel

## Abstract

Mutations in genes involved in the biosynthesis of the glycosylphosphatidylinositol (GPI) anchor cause autosomal recessive glycosylation defects, with a wide phenotypic spectrum of intellectual disability, seizures, minor facial dysmorphism, hypotonia, and elevated serum alkaline phosphatase. We now describe consanguineous Bedouin kindred presenting with an autosomal recessive syndrome of intellectual disability and elevated serum alkaline phosphatase. Genome-wide linkage analysis identified 6 possible disease-associated loci. Whole-exome sequencing followed by Sanger sequencing validation identified a single variant in* PGAP2* as the disease-causing mutation (C.554G>A; p.185(R>Q)), segregating as expected within the kindred and not found in 150 Bedouin controls. The mutation replaces a highly conserved arginine residue with glutamine within the Frag1 (FGF receptor activating) domain of PGAP2. Interestingly, this mutation is a known dbSNP variant (rs745521288, build 147) with a very low allele frequency (0.00000824 in dbSNP, no homozygotes reported), highlighting the fact that dbSNP variants should not be automatically ruled out as disease-causing mutations. We further showed that* PGAP2* is ubiquitously expressed, but in line with the disease phenotype, it is highly transcribed in human brain, skeletal muscle, and liver. Interestingly, a mild phenotype of slightly elevated serum levels of alkaline phosphatase and significant learning disabilities was observed in heterozygous carriers.

## 1. Introduction

**Figure 1:**
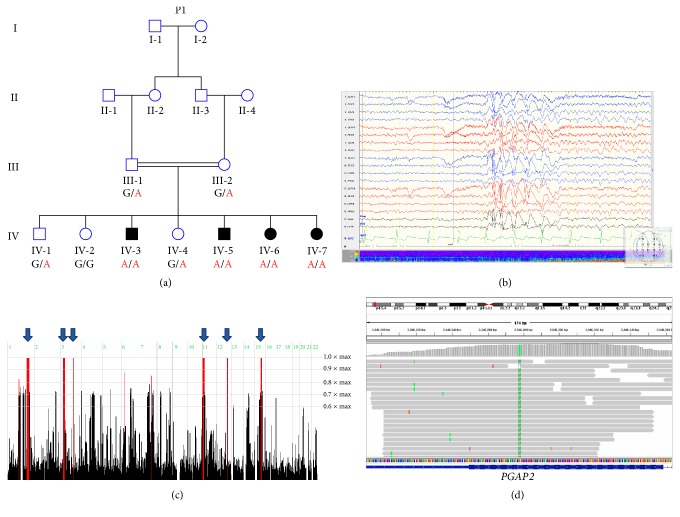
Pedigree of studied kindred, EEG recordings, homozygosity mapping, and the* PGAP2* variant: (a) pedigree of consanguineous Bedouin kindred studied. Below each individual are the alleles of the* PGAP2* mutation, whereas G (in black) represents the wild-type allele while A (in red) represents the mutant allele. (b) EEG recordings of patient IV-5 during wakefulness showing generalized interictal spike and slow epileptiform discharges more prominent anteriorly. (c) Homozygosity-Mapper plot; blue arrows present homozygous loci shared by three affected individuals. (d) Integrative Genomic Viewer (IGV) showing the* PGAP2* variant.

Biosynthesis of the glycosylphosphatidylinositol (GPI) anchor is a highly conserved process in eukaryotes. GPI-anchored proteins play an important role in membrane protein trafficking, cell-surface adhesion, and protection. Generation of GPI anchors results from a posttranslational modification of specific proteins, which is initiated by its synthesis at the endoplasmic reticulum. After synthesis, the GPI-anchor is continuously remodeled and transported to the cell membrane through the Golgi apparatus [1]. To date, mutations in six genes (*PIGV, PIGY, PIGO, PGAP2, PIGW,* and* PGAP3*) have been shown to cause hyperphosphatasia with mental retardation syndrome (HPMRS) in an autosomal recessive mode of inheritance (no autosomal dominant mutations have been reported thus far). Moreover, all of these genes are involved in GPI-anchor biosynthesis. PGAP2 is a noncatalytic protein which assists in attaching a saturated fatty acid to the anchor. This function of PGAP2 occurs in the Golgi apparatus and is required for stable association between GPI-anchored proteins and the cell-surface membrane rafts [2]. Seven different mutations in* PGAP2* have been described to cause hyperphosphatasia with mental retardation syndrome thus far [1–4]. In this study, we set out to decipher the molecular basis of apparently autosomal recessive hyperphosphatasia with mental retardation syndrome (HPMRS) in four individuals of a consanguineous Bedouin family (Figure 1(a)).

## 2. Materials and Methods

### 2.1. Subjects and Clinical Phenotyping

Nine family members of consanguineous Bedouin kindred were studied (Figure 1(a)). Clinical phenotyping was determined by an experienced team of pediatric neurologists and geneticists for all affected individuals, their parents, and siblings. DNA samples were obtained following approval of the Soroka Medical Center Internal Review Board.

### 2.2. Homozygosity Mapping

Genome-wide homozygosity mapping of three affected individuals (IV-3, IV-6, and IV-7) (Figure 1(a)) was performed using CytoScan HD Array, which includes over 2.6 million functional markers across the entire genome and is suitable for copy number variation (CNV) detection as well as for genome-wide linkage analysis (Affymetrix, Inc.). Homozygosity mapping analysis was carried out using the open online software: Homozygosity-Mapper (http://www.homozygositymapper.org/) [5]. All physical positions mentioned are according to the GRCh37/hg19 genome assembly.

### 2.3. Sequencing Analysis

Whole-exome sequencing was performed by Centogene Company (Rostock, Germany), with 70–100x average coverage (~95% targeted bases covered >10x). Library preparation was done using Nextera® Rapid Capture Exome Kit and sequencing was performed with Hiseq 2000 sequencing system (Illumina, Inc.). Data were analyzed using QIAGEN's Ingenuity® Variant Analysis™ software (https://www.qiagen.com/ingenuity) from QIAGEN Redwood City as previously described [6]. Using their filtering cascade, we excluded variants that are observed with an allele frequency greater than or equal to 1.0% of the genomes in the 1000 genomes project, NHLBI ESP exomes (All), or the Allele Frequency Community. In addition, we excluded variants which appeared in a homozygous state in our in-house whole-exome sequencing database of 150 Bedouin control samples. Furthermore, we kept variants which are predicted to have a deleterious effect upon protein coding sequences (e.g., frameshift, in-frame indel, stop codon change, missense or predicted to disrupt splicing by MaxEnt Scan) and variants which were experimentally observed to be associated with a phenotype: pathogenic, possibly pathogenic, or disease-associated according to the Human Gene Mutation Database (HGMD). Following the above filtering, of the remaining variants we selected only homozygous variants which were located within any of the homozygous loci that were identified by genome-wide homozygosity mapping. Validation and segregation analysis of the* PGAP2* mutation was done via Sanger sequencing using the following primers: forward 5′- GCCCATTCCCTAGGATCGC -3′; reverse 5′- AACACAATGGCAGCCAGTCC -3′ (225 bp amplicon). Annealing temperature used was 60°C, the extension time was set for 30 seconds, and PCR repeats were set for 40 cycles.

### 2.4. Multiple Sequence Alignment

Eight representative* PGAP2* orthologues were selected for multiple sequence alignment (MSA). All protein sequences were taken from the National Center for Biotechnology Information GenBank (http://www.ncbi.nlm.nih.gov). The RefSeq sequence accession numbers for* D. rerio*,* X. tropicalis*,* R. norvegicus*,* M. musculus*,* P. troglodytes*,* B. taurus*,* H. sapiens,* and* C. lupus familiaris* of PGAP2 orthologues used for the analysis are NP_001013562.1, NP_001106477.1, XP_006229899.1, XP_006507706.1, XP_001159411.2, NP_001092581.1, NP_001243169.1, and XP_005633631.1, respectively. Protein MSA was performed using Clustal Omega program (http://www.ebi.ac.uk/Tools/msa/clustalo/) [7].

### 2.5. *PGAP2* Expression Analysis

A panel of cDNA samples was prepared from total RNA derived of 21 normal human tissues (Clontech Laboratories, Inc.), using Verso cDNA kit (Thermo Scientific™). Two sets of PCR primers were designed to amplify cDNA rather than genomic DNA of human* PGAP2* and of glyceraldehyde 3-phosphate dehydrogenase* (GAPDH)* housekeeping gene as a control. Primers used for* PGAP2* amplification (237 bp amplicon) are as follows: forward 5′- AAACAGCGGCTCTTCATCAT -3′; reverse 5′- CAAGCAGGACTGAAGGGTTC -3′; primers used for* GAPDH* (452 bp amplicon) are as follows: forward 5′- ACCACAGTCCATGCCATCAC -3′; reverse 5′- TCCACCACCCTGTTGCTGT -3′. Annealing temperature used was 58°C, the extension time was set for 30 seconds, and the PCR reaction repeats were of 35 cycles for both reactions.

## 3. Results

### 3.1. Clinical Characterization

Four individuals of consanguineous Bedouin family (Figure 1(a)) presented with a syndrome of intellectual disability and elevated serum alkaline phosphatase (Table 1). All affected individuals were born at term with normal birth weights, following uneventful pregnancies. There were no facial dysmorphism or any further abnormalities on physical examination of all patients, including thorough neurological examination, ruling out cranial, motor, sensory, gait, and cerebellar function abnormalities. Brain magnetic resonance imaging (MRI) of patients demonstrated no specific abnormal findings. Electroencephalogram (EEG) recordings done for patient IV-5 during wakefulness showed generalized interictal spike and slow epileptiform discharges that were more prominent anteriorly (Figure 1(b)). Karyotype and chromosomal microarrays (CMA) as well as molecular testing for fragile X syndrome were normal as well (Table 1).

### 3.2. Genetic Analysis

Genome-wide homozygosity mapping analysis of samples derived from three affected individuals (IV-3, IV-6, and IV-7) identified 6 different disease-associated loci (Table 2 and Figure 1(c)). Of the six genes previously shown to cause hyperphosphatasia with mental retardation syndrome (HPMRS), only one lies within any of these homozygous loci:* PGAP2*, located at position chr11:3,819,049-3,847,601, residing within the chromosome 11 locus (Table 2).

Whole-exome sequencing data of individuals IV-3 (Figure 1(a)) were filtered for normal variants as described in Materials and Methods. Following the above filtering, four different rare variants remained: a c.3980A>G, p.(N1327S) in* RFX*, a c.277A>G, p.(I93V) in* OR10A5*, a c.1133G>A, p.(R323Q) in* TUB,* and a c.554G>A, p.(Arg185Gln) in* PGAP2*, a gene previously associated with the disease phenotype (Figure 1(d)). These four potentially pathogenic variants which remained after the filtration cascade and which were located within the homozygous loci identified by genome-wide homozygosity mapping were further assayed through segregation analysis (Table 3).

Only a single homozygous variant was found to reside within one of the above loci and to fully segregate as expected in autosomal recessive heredity (via Sanger sequencing) within all family members (i.e., parents were obligatory carriers, unaffected siblings were either carriers or homozygous for the wild-type allele, and affected individuals were all homozygous for the mutated allele; data not shown).

**Figure 2:**
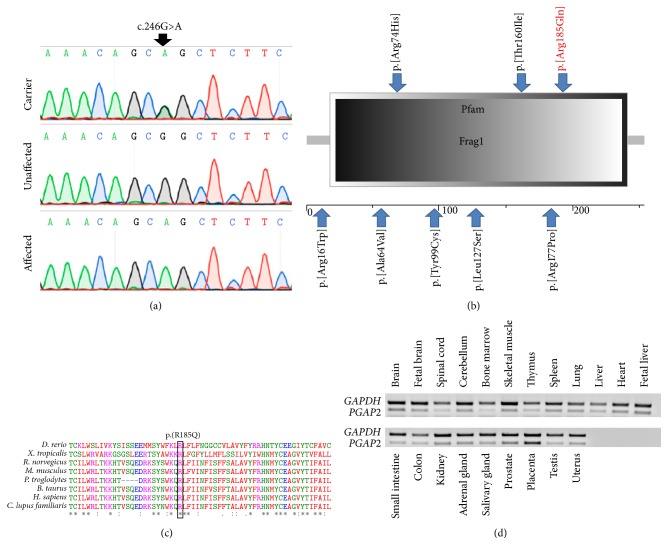
The* PGAP2* mutation, domain architecture, conservation, and expression pattern: (a) Sanger sequencing of an obligatory carrier (III-1), an affected individual (IV-3), and an unaffected individual (IV-2). (b) The predicted domain architecture of PGAP2, showing its Frag1 domain. The p.(Arg185Gln) mutation is marked in red. Blue arrows highlight additional* PGAP2* mutations previously shown to cause hyperphosphatasia with mental retardation syndrome (HPMRS). (c) Multiple sequence alignment of selected* PGAP2* orthologues. The mutation is predicted to cause p.(Arg185Gln) (boxed in black) at a highly conserved arginine residue within the putative Frag1 domain of PGAP2. (d) RT-PCR of 21 normal human tissues demonstrating the expression pattern of* PGAP2*.

The mutation found is c.554G>A, p.(Arg185Gln) in* PGAP2*, within the chromosome 11 locus (Figures 1(a) and 2(a)). The mutation resides within the Frag1 domain of the mature PGAP2 encoded protein (Figure 2(b)) and is predicted to replace a highly conserved arginine residue with a glutamine residue (Figure 2(c)). The mutation was also found to be possibly damaging by Polyphen-2 predictions with a score of 0.942 (http://genetics.bwh.harvard.edu/pph2/). The* PGAP2* variant has been previously reported in the dbSNP database (https://www.ncbi.nlm.nih.gov/projects/SNP/) and in the Exome Aggregation Consortium (ExAC) Browser (http://exac.broadinstitute.org/) with an allele frequency of 0.000008122 (two carriers out of 246258 individuals; no homozygotes reported). In fact, there are no* PGAP2* homozygous loss-of-function (LoF) mutations (stop gain, frameshift, or essential splice site mutations) or missense mutations which are reported in the Exome Aggregation Consortium (ExAC). The* PGAP2* transcript has multiple splicing isoforms and is highly conserved throughout evolution. Screening of the mutation in 150 ethnically matched controls (300 chromosomes) identified no carriers and no homozygous mutants (data not shown).

### 3.3. *PGAP2* Expression Patterns

Analysis of* PGAP2* expression in various normal human tissues by RT-PCR demonstrated that it is ubiquitous. However, while* PGAP2* is moderately expressed in most tissues examined, it is highly transcribed in brain, cerebellum, skeletal muscle, heart, fetal liver, and placenta (Figure 2(d)).

## 4. Discussion

The kindred studied here, as is most of the Bedouin community in southern Israel, originates from Saudi Arabia. This community is unique in its high prevalence of consanguineous marriages, high birth rate, and extreme inbreeding within clans, leading to high incidence of monogenic diseases [8]. Here we describe an autosomal recessive form of hyperphosphatasia with mental retardation syndrome (HPMRS). Homozygosity mapping analysis of samples derived from three affected individuals identified 6 different disease-associated loci (Figure 1(c)). Amongst the known genes associated with HPMRS,* PGAP2* was the only gene residing within any of the disease-associated loci identified by homozygosity mapping analysis. Exome sequencing combined with Sanger sequencing validation and segregation analysis identified a* PGAP2 *mutation as the only probable cause for this syndrome: c.554G>A (NM_001256240.1), p.(Arg185Gln) (Figures 1(d) and 2(a)). The mutation segregated within the kindred as expected for autosomal recessive heredity (Figure 1(a)) and was not found in a homozygous or heterozygous state in 150 ethnically matched controls (data not shown). Clustal Omega analysis (http://www.ebi.ac.uk/Tools/msa/clustalo/) [7] showed that the p.(Arg185Gln) mutation in PGAP2 replaces a highly conserved arginine residue with glutamine within Frag1 domain (Figures 2(b) and 2(c)). Six different mutations within the Frag1 domain have been previously described [1–4] (Figure 2(b)). All patients with mutations in this domain suffer from psychomotor retardation, intellectual disability, hypotonia, and increased serum alkaline phosphatase. While most features of this syndrome are shared between individuals with mutations in the PGAP2 Frag1 domain, some phenotypic variability exists, with various degrees of microcephaly, seizures, and mild dysmorphic features. All our patients, homozygous for the same mutation, have in common all the basic features of the phenotype, yet have variable penetrance of seizures with no microcephaly or dysmorphism.

PGAP2 has multiple coding and noncoding isoforms. All coding transcripts harbor the c.554G>A mutation. We demonstrated that* PGAP2* is highly transcribed in the human brain, skeletal muscle, heart, and fetal liver (Figure 2(d)) in line with the disease phenotype. PGAP2 is a noncatalytic highly conserved protein which assists in attaching a saturated fatty acid in the biosynthesis of glycosylphosphatidylinositol (GPI) anchor. Mutations in six different genes (*PIGV*,* PIGY*,* PIGO*,* PGAP2*,* PIGW,* and* PGAP3*) involved in GPI-anchor biosynthesis have been shown to cause hyperphosphatasia with mental retardation syndrome (HPMRS). All HPMRS cases described to date, regardless of the specific mutated gene, are inherited in an autosomal recessive fashion. To date, 7 mutations in* PGAP2* gene have been identified as a cause of hyperphosphatasia with mental retardation syndrome (HPMRS) (Figure 2(b)).

Interestingly, we show that two carriers (IV-1 and IV-4) also presented slightly high levels of alkaline phosphatase in serum and have also been reported to have significant learning disabilities, though without mental retardation (Table 1). In contrast, their sibling (IV-2) which is homozygous for the wild-type allele reported to have high academic performances (having completed a university Master's degree). It would be of interest to examine whether similar cases of a mild phenotype have been reported for other heterozygous individuals, carrying different mutations in* PGAP2*.

Altogether, our data demonstrate that the autosomal recessive syndrome of hyperphosphatasia with mental retardation in this family is caused by a rare homozygous variant in* PGAP2* which was previously reported in dbSNP database and that a mild phenotype could be observed in heterozygous carriers.

## Figures and Tables

**Table 1:** Patients' clinical data.

Patient	Hx	Physical exam	Lab measurements	Imaging + EEG
Patient 1 25-year-old male sibling IV-3	Mild mental retardation Mood problems, depression Speech difficulties Past history of febrile convulsions	No organ anomaly No signs of dysmorphism Normal cranial, motor, sensory, gait, and cerebellar exam	Elevated alkaline phosphatase: 499 IU/L at 23 yrs of age	Normal brain CTA Normal EEG

Patient 2 18-year-old male sibling IV-5	Developmental delay Moderate mental retardation Behavioral problems- aggression Past history of seizures without fever	No organ anomaly No signs of dysmorphism Normal cranial, motor, sensory, gait, and cerebellar exam	Elevated alkaline phosphatase: 673 IU/L at 17 yrs of age	Normal brain MRI EEG: normal background activity Generalized spike and wave interictal epileptiform discharges

Patient 3 15-year-old female sibling IV-6	Mild mental retardation Mood problems- depression Speech difficulties	No organ anomaly No signs of dysmorphism	Elevated alkaline phosphatase: >1000 IU/L at 10 yrs of age	

Patient 4 11-year-old female sibling IV-7	Developmental delay Mild mental retardation Speech difficulties Enuresis	No organ anomaly No signs of dysmorphism Normal cranial, motor, sensory, gait, and cerebellar exam	Elevated alkaline phosphatase: 1318 IU/L at 10 yrs of age	Normal spinal MRI

Carrier 1 22-year-old male sibling IV-1	Reported Learning disabilities without mental retardation	No organ anomaly No signs of dysmorphism Normal cranial, motor, sensory, gait, and cerebellar exam	Mildly elevated alkaline phosphatase: 122 IU/L at 20 yrs of age	Normal brain CT scan

Carrier 2 19-year-old female sibling IV-4	Reported learning disabilities without mental retardation	No organ anomaly No signs of dysmorphism Normal cranial, motor, sensory, gait, and cerebellar exam	Mildly elevated alkaline phosphatase: 134 IU/L at 18 yrs of age	—

Homozygous wild-type 24-year-old female sibling IV-2	High academic performance	No organ anomaly No signs of dysmorphism Normal cranial, motor, sensory, gait, and cerebellar exam	Normal alkaline phosphatase: 84 IU/L at 23 yrs of age	Normal brain CT

Patients IDs correspond with pedigree in Figure 1(a). Hx = medical history; IU/L = international units per liter.

**Table 2:** Loci identified by homozygosity mapping.

Chromosome	SNPs defining the borders of loci	Genomic positions (GRCh37/hg19)	Loci length (Mb)
1	rs7534216–rs2070257	179627385–202117002	~22.5
3	rs7644408–rs2276868	29891885–40498845	~10.6
3	rs9876197–rs13094803	119099303–123565063	~4.5
11	rs3852527–rs16906385	2826603–20552960	~17.8
12	rs4762315–rs35735	97055302–100950371	~3.1
15	rs2251480–rs1126308	54167341–63429086	~9.2

**Table 3:** Candidate variants within homozygous loci.

Gene	Genomic position	Transcript variant	Protein variant	dbSNP ID
*RFX*	Chr15:56,382,731-56,535,483	c.3980A>G	p.(N1327S)	—
*OR10A5*	Chr11:6,866,914-6,867,867	c.277A>G	p.(I93V)	rs540522650
*TUB*	Chr11:8,060,180-8,127,654	c.1133G>A	p.(R323Q)	rs749889465
*PGAP2*	Chr11:3,819,049-3,847,601	c.554G>A	p.(R185Q)	rs745521288

## Abbreviations

MRI: Magnetic resonance imaging
CT: Computed tomography
CTA: Computed tomography angiography
RT-PCR: Reverse transcription polymerase chain reaction
Mb: Million bases
ESP: Exome Sequencing Project
NHLBI: National Heart, Lung, and Blood Institute
NCBI: National Center for Biotechnology Information
WT: Wild-type
IRB: Institutional review board
MSA: Multiple sequence alignment
HGMD: Human gene mutation database
SNP: Single nucleotide polymorphism
ExAC: Exome Aggregation Consortium
IGV: Integrative Genomic Viewer
HPMRS: Hyperphosphatasia with mental retardation syndrome
ALP: Alkaline phosphatase.

## Additional Points

*Web Resources.* The following resources were used: UCSC genome browser: https://genome.ucsc.edu/; Chromas: http://technelysium.com.au/; HaploPainter: http://haplopainter.sourceforge.net/index.html; Primer3 (V.0.4.0): http://frodo.wi.mit.edu/primer3/; SNP database: https://www.ncbi.nlm.nih.gov/projects/SNP/; Clustal Omega software: http://www.ebi.ac.uk/Tools/msa/clustalo/; NHLBI Exome Sequencing Project, Seattle, WA: http://evs.gs.washington.edu/EVS/; Online Mendelian Inheritance in Man (OMIM): http://www.omim.org/; Biotechnology Information GenBank: https://www.ncbi.nlm.nih.gov/; Homozygosity-Mapper: http://www.homozygositymapper.org/; 1000 genomes project: http://www.1000genomes.org/; PolyPhen-2: http://genetics.bwh.harvard.edu/pph2/; ExAC Browse: http://exac.broadinstitute.org/.
